# Development of an artificial intelligence bacteremia prediction model and evaluation of its impact on physician predictions focusing on uncertainty

**DOI:** 10.1038/s41598-023-40708-2

**Published:** 2023-08-19

**Authors:** Dong Hyun Choi, Min Hyuk Lim, Ki Hong Kim, Sang Do Shin, Ki Jeong Hong, Sungwan Kim

**Affiliations:** 1 Department of Biomedical Engineering, Seoul National University College of Medicine, Seoul, South Korea; 2 Transdisciplinary Department of Medicine and Advanced Technology, Seoul National University Hospital, Seoul, South Korea; 3 Innovative Medical Technology Research Institute, Seoul National University Hospital, Seoul, South Korea; 4 Institute of Medical and Biological Engineering, Seoul National University, Seoul, South Korea; 5 Department of Emergency Medicine, Seoul National University Hospital, Seoul, South Korea; 6 Department of Emergency Medicine, Seoul National University College of Medicine, Seoul, South Korea; 7 Laboratory of Emergency Medical Services, Seoul National University Hospital Biomedical Research Institute, Seoul, South Korea; 8 Institute of Bioengineering, Seoul National University, Seoul, South Korea

**Keywords:** Bacterial infection, Machine learning, Diagnosis

## Abstract

Prediction of bacteremia is a clinically important but challenging task. An artificial intelligence (AI) model has the potential to facilitate early bacteremia prediction, aiding emergency department (ED) physicians in making timely decisions and reducing unnecessary medical costs. In this study, we developed and externally validated a Bayesian neural network-based AI bacteremia prediction model (AI-BPM). We also evaluated its impact on physician predictive performance considering both AI and physician uncertainties using historical patient data. A retrospective cohort of 15,362 adult patients with blood cultures performed in the ED was used to develop the AI-BPM. The AI-BPM used structured and unstructured text data acquired during the early stage of ED visit, and provided both the point estimate and 95% confidence interval (CI) of its predictions. High AI-BPM uncertainty was defined as when the predetermined bacteremia risk threshold (5%) was included in the 95% CI of the AI-BPM prediction, and low AI-BPM uncertainty was when it was not included. In the temporal validation dataset (N = 8,188), the AI-BPM achieved area under the receiver operating characteristic curve (AUC) of 0.754 (95% CI 0.737–0.771), sensitivity of 0.917 (95% CI 0.897–0.934), and specificity of 0.340 (95% CI 0.330–0.351). In the external validation dataset (N = 7,029), the AI-BPM’s AUC was 0.738 (95% CI 0.722–0.755), sensitivity was 0.927 (95% CI 0.909–0.942), and specificity was 0.319 (95% CI 0.307–0.330). The AUC of the post-AI physicians predictions (0.703, 95% CI 0.654–0.753) was significantly improved compared with that of the pre-AI predictions (0.639, 95% CI 0.585–0.693; p-value < 0.001) in the sampled dataset (N = 1,000). The AI-BPM especially improved the predictive performance of physicians in cases with high physician uncertainty (low subjective confidence) and low AI-BPM uncertainty. Our results suggest that the uncertainty of both the AI model and physicians should be considered for successful AI model implementation.

## Introduction

Recent progress in mathematical algorithms and computing power has led to a rapid growth in the development of artificial intelligence (AI) models within the healthcare industry^1^. The utilization of multi-modal data, including structured, text, and image data, along with advanced algorithms, has resulted in significant improvements in the performance of AI models^2,3^. Despite an abundance of evidence from retrospective studies that AI models outperform or perform equally to human experts, few have been deployed in the field^4^. One reason for this is physicians’ lack of trust in AI algorithms due to their “black box” nature. Another important reason for the poor adoption is that most previous research did not consider the interaction between the AI system and its user but regarded the system as an autonomous agent^5^. As long as physicians make the final decision, an AI model will be used as a clinical decision support system (CDSS). Therefore, the performance of a physician and an AI model working in tandem may not be equivalent to that assessed in retrospective studies^6^.

Uncertainty is one of the key elements of the medical decision-making process. Arriving at a medical decision requires reducing uncertainty by acquiring information through history taking, diagnostic tests, and possibly AI model predictions^7^. When a physician is uncertain about a decision, they may seek more information, including AI model prediction results. However, if a prediction from an AI model shows high uncertainty or is difficult to interpret, the physician may not accept the results^8^. Therefore, the uncertainty of both the physician and the AI model can affect the physician–AI interaction during the decision-making process. Some recent studies have concluded that uncertainty-informed AI models, such as Bayesian neural network (BNN)-based models, achieve superior performance compared with AI models that did not consider their uncertainty^9,10^. However, research on physician response to an AI CDSS considering the uncertainty of both the AI and physician is limited^11^.

Bacteremia, which refers to the presence of bacteria in the bloodstream, is a major public health burden with high incidence and mortality rates of 113–204 and 20.4–37.8 per 100,000 person-years, respectively^12^. Blood cultures, which are essential for diagnosing bacteremia and revealing the causative organisms, are frequently performed in emergency department (ED) patients with suspected infection. Because the consequences of undetected bacteremia can be fatal, ED physicians tend to perform blood cultures even in low-risk patients^13^. Additionally, ED physicians often order blood cultures before checking the results of diagnostic tests because early suspicion and antibiotic administration reduces mortality in patients with bacteremia, and the sensitivity of blood cultures decreases after antibiotic administration^14–16^. Consequently, blood cultures are overused, have low yields (7.5–15%), and show high contamination rates^13,17^. A previous study reported the cost of blood collection per patient to range from $96 to $423, with additional expenses incurred for patients with blood culture contamination due to unnecessary treatments^18^.

Previous studies have attempted to address this concern by developing bacteremia prediction models to identify low- and high-risk patients^19–22^. Ideally, these models can be used to avoid blood cultures for predicted low-risk patients and initiate early antibiotic treatment for high-risk patients. Moreover, since blood culture results usually take more than 24 h to be reported, bacteremia prediction models can be valuable tools for assisting ED physicians in making timely medical decisions^23^. While several traditional score-based and machine learning models have been developed, they are rarely applied in the field due to unsatisfactory performance, lack of trust, lack of perceived utility, and limited usability (most existing models include laboratory test results as their input)^13,24^. The decision to perform blood cultures still relies on physician gestalt, which was shown in a recent study to have comparable discrimination performance in predicting bacteremia to that of existing prediction models^13^. Despite the remaining risk for non-accurate predictions, AI-based models, with their superior performance, hold promise in providing valuable assistance and reducing medical costs. However, it remains unknown whether an AI bacteremia prediction model used as a CDSS will indeed enhance physicians' predictive abilities.

In this study, we developed and externally validated a BNN-based AI bacteremia prediction model (AI-BPM). Additionally, we evaluated the impact of AI-BPM on the predictive performance of physicians using historical patient data and determined the factors that influence physician response to the AI. We hypothesized that physician predictive performance will improve after observing AI-BPM predictions, and this prediction change will be associated with the uncertainty of both the physician and the model.

## Results

**Figure 1:**
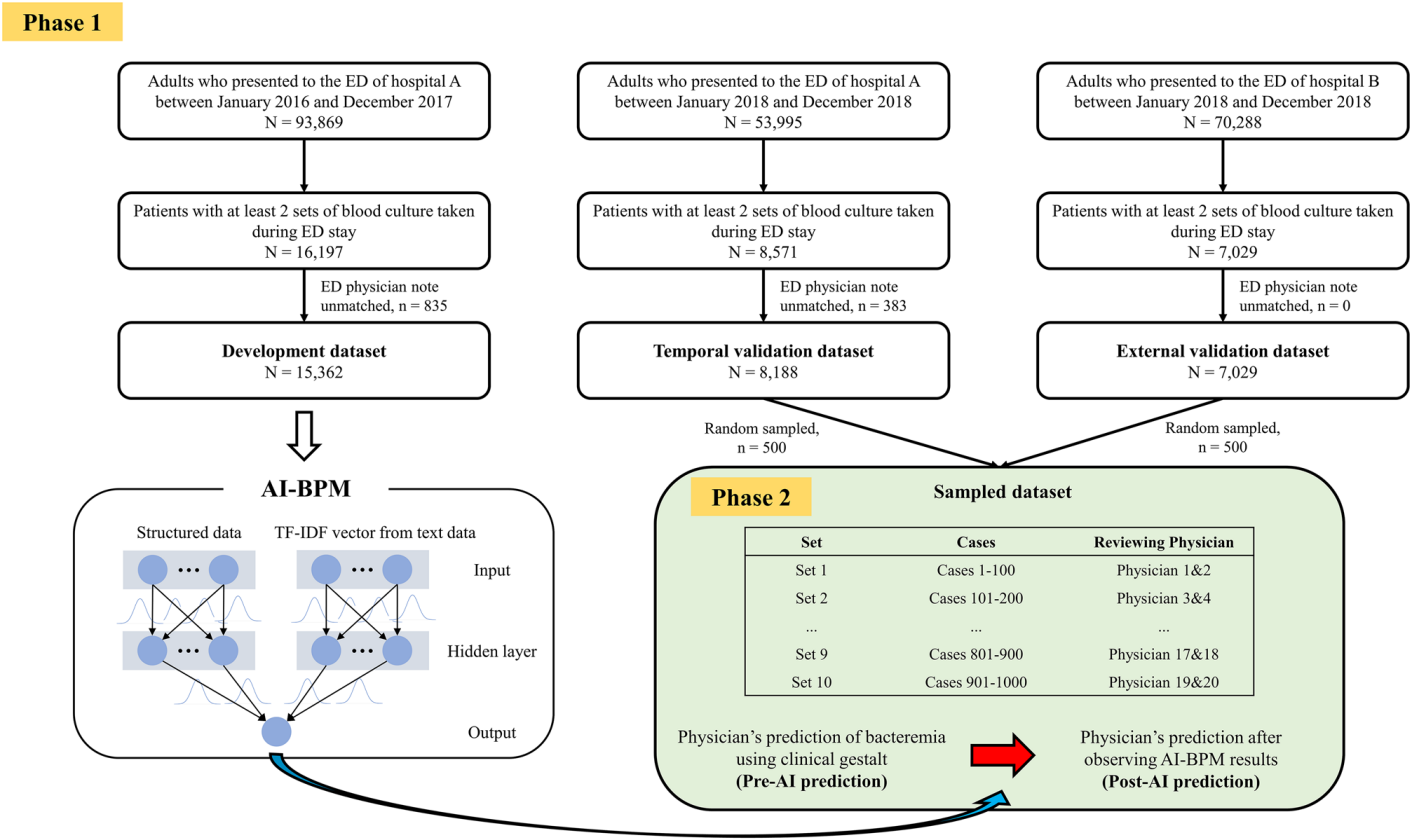
Overall study flow diagram. ED, emergency department; TF-IDF, term frequency-inverse document frequency; AI, artificial intelligence.

This research was conducted in two phases: In Phase 1, we performed a retrospective cohort study using data from two academic tertiary hospitals for the AI-BPM development, temporal validation, and external validation. In Phase 2, the performance of physicians in predicting bacteremia before and after the use of AI-BPM was evaluated (Fig. 1).

### Phase 1: Development and validation of AI-BPM

Adult (aged ≥ 18 years) ED patients, who had at least two sets of blood cultures taken during their ED stay, were included for analysis. 15,362, 8,188, and 7,029 cases were included in the development, temporal validation, and external validation dataset, respectively, with mean ages ranging 62.3–65.6 years and proportion of females ranging 45.1–45.8%. The proportion of patients with bacteremia were 10.9%, 10.3%, and 13.6% in the development, temporal validation, and external validation datasets, respectively (Table 1). In the development dataset, patients with bacteremia were older, more likely to use an ambulance, and less likely to be referred from other hospitals than patients without bacteremia; they also exhibited lower blood pressure, higher heart rate (HR), and higher body temperature (BT). Bacteremia patients were more likely to have a history of chills, vomiting, and abdominal pain (Supplementary Table 1).

**Table 1:** Characteristics and outcomes of patients in each dataset.

	Development dataset	Temporal validation dataset	External validation dataset
	(N = 15,362)	(N = 8,188)	(N = 7,029)
Demographics			
Age, years	62.3 (15.9)	63.4 (16.0)	65.6 (17.3)
Sex, female	6,929 (45.1)	3,738 (45.7)	3,218 (45.8)
Ambulance use	5,496 (35.8)	3,100 (37.9)	2,436 (34.7)
Referred from other hospital	3,665 (23.9)	2,040 (24.9)	1,791 (25.5)
Injury related visit	135 (0.9)	49 (0.6)	99 (1.4)
ED triage level			
Level 1	791 (5.1)	481 (5.9)	126 (1.8)
Level 2	4,017 (26.1)	2,041 (24.9)	1,048 (14.9)
Level 3	8,972 (58.4)	4,575 (55.9)	4,439 (63.2)
Level 4	1,566 (10.2)	1,026 (12.5)	1,312 (18.7)
Level 5	16 (0.1)	65 (0.8)	104 (1.5)
Initial mental status			
Alert	13,894 (90.4)	7,311 (89.3)	6,167 (87.7)
Verbal	1,073 (7.0)	572 (7.0)	274 (3.9)
Pain	285 (1.9)	231 (2.8)	523 (7.4)
Unresponsive	110 (0.7)	74 (0.9)	65 (0.9)
Initial vital signs			
SBP, mmHg	136.1 (28.2)	137.2 (29.8)	128.9 (25.9)
DBP, mmHg	76.5 (15.1)	77.1 (15.7)	70.8 (16.2)
HR, mmHg	99.6 (20.1)	99.7 (20.8)	98.3 (20.5)
RR, mmHg	19.8 (4.4)	19.9 (4.4)	19.8 (4.6)
BT, °C	37.4 (1.1)	37.4 (1.1)	37.6 (1.1)
Symptom history			
Chills	3,792 (24.7)	2,164 (26.4)	1,065 (15.2)
Vomiting	1,598 (10.4)	767 (9.4)	379 (5.4)
Abdominal pain	2,631 (17.1)	1,087 (13.3)	733 (10.4)
Outcomes			
Bacteremia	1,670 (10.9)	847 (10.3)	957 (13.6)
Contamination	233 (1.5)	125 (1.5)	128 (1.8)
Hospital admission	8,912 (58.0)	3,564 (43.5)	4,562 (64.9)
Death in ED	38 (0.2)	21 (0.3)	52 (0.7)

Categorical variables are presented as numbers (proportions) and continuous variables are presented as means (standard deviations). Hospital admission included patients admitted to the ward or intensive care unit. Abbreviations: ED, emergency department; SBP, systolic blood pressure; DBP, diastolic blood pressure; HR, heart rate; RR, respiratory rate; BT, body temperature.

**Figure 2:**
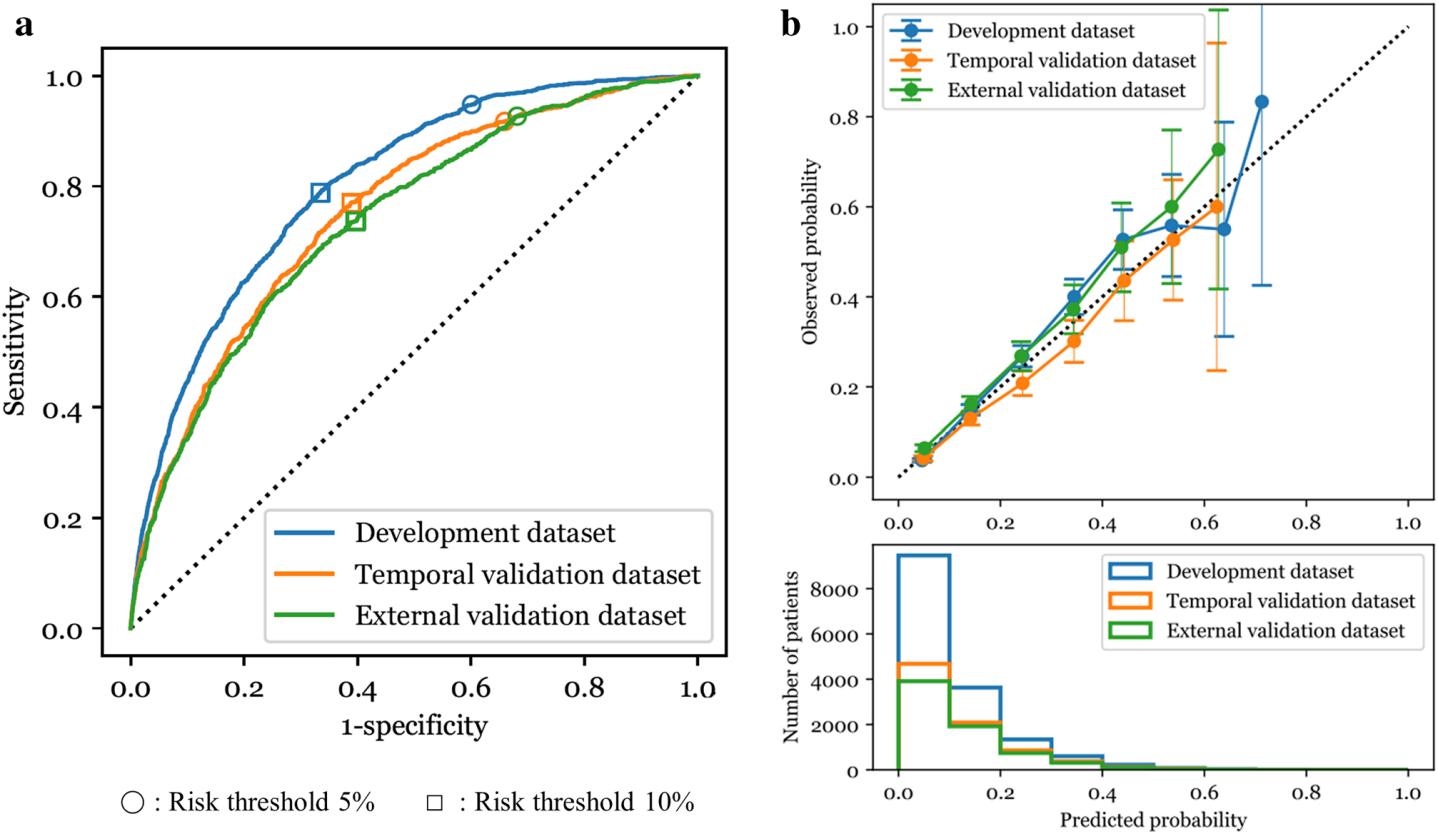
(**a**) Receiver operating characteristic curve and (**b**) calibration plot for the AI-BPM bacteremia prediction. The 95% confidence intervals are drawn as error bars at each point of the calibration plot.

The area under the receiver operating characteristic curves (AUCs) (95% confidence intervals (CIs)) of the AI-BPM were 0.804 (0.793–0.814), 0.754 (0.737–0.771), and 0.738 (0.722–0.755) in the development, temporal validation, and external validation datasets, respectively (Fig. 2a). The AI-BPM showed suitable calibration in all datasets, as shown in Fig. 2b. When the bacteremia risk threshold was set to 5%, the sensitivity and specificity of the AI-BPM in the external validation dataset were 0.927 (0.909–0.942) and 0.319 (0.307–0.330), respectively, and at a threshold level of 10%, they were 0.737 (0.708–0.764) and 0.603 (0.591–0.615), respectively (Table 2). The top 20 important features of the AI-BPM (global feature importance) are shown in Supplementary Fig. 1.

**Table 2:** Discrimination performance of the AI-BPM for predicting bacteremia.

Dataset	AUC (95% CI)	Sensitivity (95% CI)	Specificity (95% CI)	PPV (95% CI)	NPV (95% CI)
Risk threshold: 5%					
Development	0.804 (0.793–0.814)	0.948 (0.936–0.958)	0.399 (0.391–0.407)	0.161 (0.154–0.169)	0.984 (0.981–0.987)
Temporal validation	0.754 (0.737–0.771)	0.917 (0.897–0.934)	0.340 (0.330–0.351)	0.138 (0.130–0.148)	0.973 (0.966–0.978)
External validation	0.738 (0.722–0.755)	0.927 (0.909–0.942)	0.319 (0.307–0.330)	0.177 (0.166–0.187)	0.965 (0.956–0.972)
Risk threshold: 10%					
Development	0.804 (0.793–0.814)	0.788 (0.768–0.807)	0.666 (0.658–0.673)	0.223 (0.213–0.234)	0.963 (0.959–0.966)
Temporal validation	0.754 (0.737–0.771)	0.770 (0.740–0.797)	0.611 (0.600–0.622)	0.186 (0.173–0.199)	0.958 (0.952–0.964)
External validation	0.738 (0.722–0.755)	0.737 (0.708–0.764)	0.603 (0.591–0.615)	0.226 (0.212–0.241)	0.936 (0.927–0.943)

AUC, area under the receiver operating characteristic curve; CI, confidence interval; PPV, positive predictive value; NPV, negative predictive value.

In the ablation study, we observed inferior performance when using only structured data or unstructured data to predict bacteremia compared to the AI-BPM, which utilized both types of data. Specifically, when only structured data was used, the AUCs (CIs) were 0.703 (0.684–0.721) and 0.679 (0.660–0.697) in the temporal validation and external validation datasets, respectively. Similarly, when only unstructured data was used, the AUCs (CIs) were 0.679 (0.660–0.698) and 0.681 (0.663–0.699) in the temporal validation and external validation datasets, respectively (Supplementary Table 2).

### Phase 2: Physician predictive performance before and after the use of AI-BPM

Five hundred cases from each of the temporal and external validation datasets were randomly sampled to construct the sampled dataset with 1,000 unique cases. The sampled dataset was then divided into ten sets, each with 100 unique cases. Twenty board-certified emergency medicine physicians were recruited to review one of the ten sets and predict the probability of bacteremia before and after observing the AI-BPM predictions for each case. Therefore, a single set was reviewed separately by two physicians, and each physician reviewed 100 cases (Fig. 1). Among the 20 reviewing physicians, 14 were currently affiliated in a tertiary hospital and 6 in a secondary hospital. The physicians had 4–10 years of experience in the ED.

**Figure 3:**
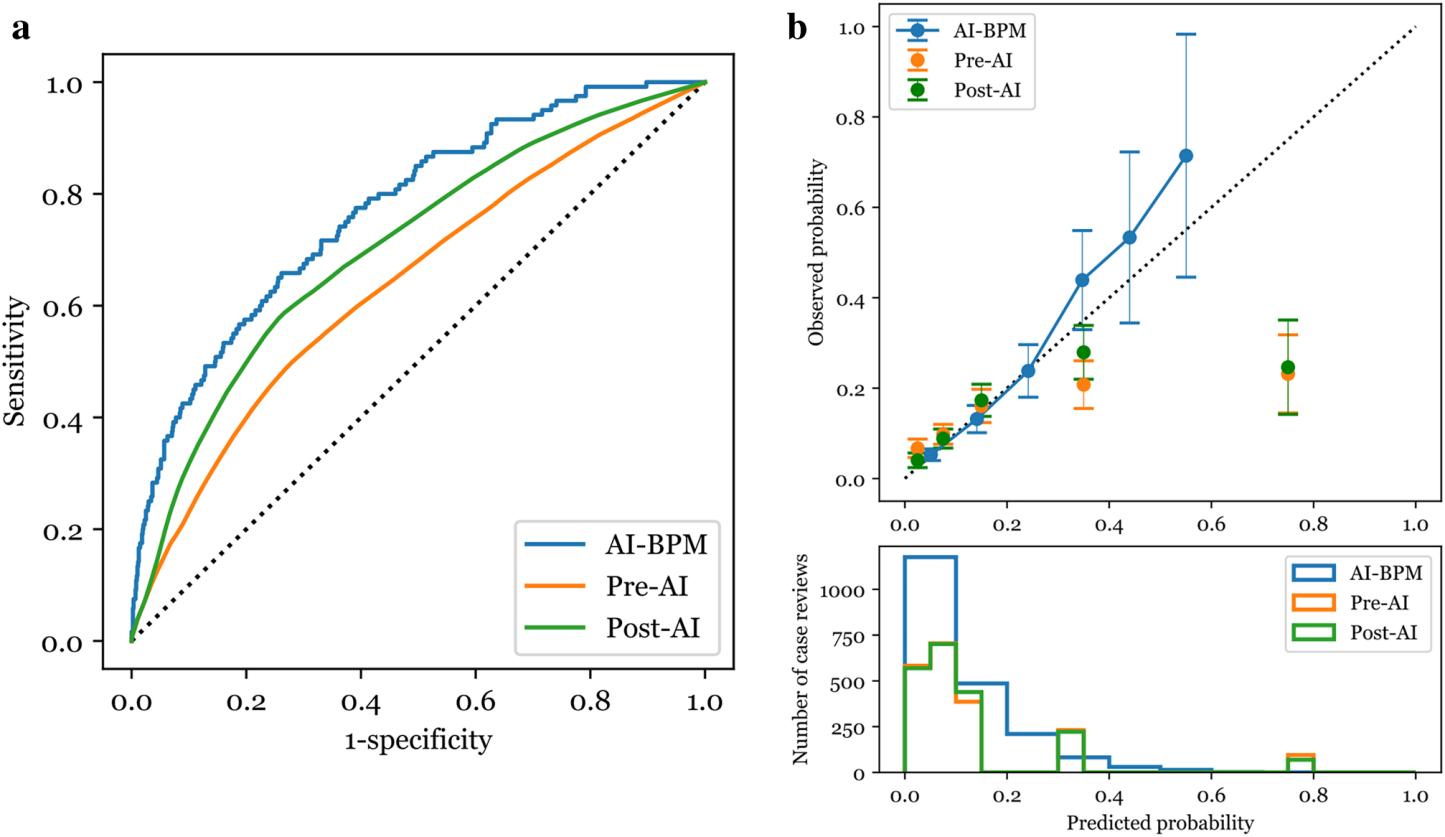
(**a**) Receiver operating characteristic curve and (**b**) calibration plot for the AI-BPM, pre-AI, and post-AI bacteremia prediction. The total number of case reviews is 2,000 since each of the 1,000 cases are reviewed twice by two different physicians. The 95% confidence intervals are drawn as error bars at each point of the calibration plot. AI, artificial intelligence.

**Figure 4:**
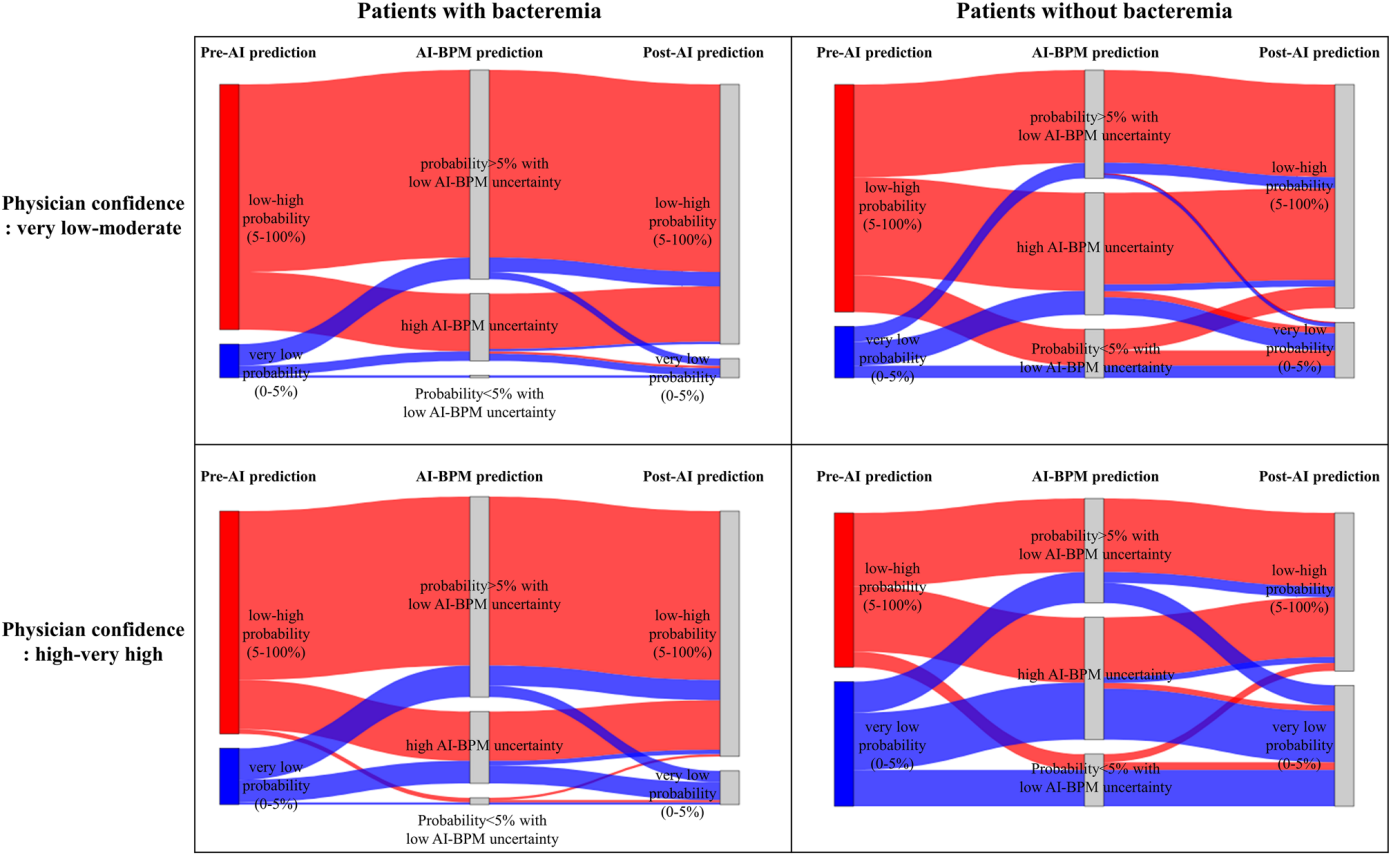
Sankey diagrams illustrating the change in physician predictions according to the physician confidence level and AI-BPM prediction result. The widths of the links are proportional to the number of case reviews corresponding to the link. Case reviews with a pre-AI prediction of low–high probability are shown as red, while case reviews with a pre-AI prediction of very low probability are shown as blue.

Among the 1,000 cases in the sampled dataset (mean age 64.1 years with a standard deviation (SD) of 14.4; female, 44.8%), the proportion of cases with bacteremia was 12.0%. The AUC of the AI-BPM in the sampled dataset was 0.770 (95% CI 0.726–0.815). The AUC of the post-AI predictions (0.703, 95% CI 0.654–0.753) was significantly improved compared with the pre-AI predictions (0.639, 95% CI 0.585–0.693; p-value < 0.001; Fig. 3). The sensitivity for the post-AI predictions (0.904, 95% CI 0.858–0.950) was also significantly increased compared with the pre-AI predictions (0.839, 95% CI 0.759–0.920; p-value = 0.02). However, no significant difference in the specificity between the pre-AI predictions (0.309, 95% CI 0.252–0.365) and post-AI predictions (0.310, 95% CI 0.262–0.358; p-value = 0.92) was observed. For cases in which the AI-BPM predicted with low uncertainty, the AUC was significantly increased for the post-AI predictions (0.710, 95% CI 0.661–0.759) compared with the pre-AI predictions (0.649, 95% CI 0.594–0.704; p-value < 0.001). In the subgroup with high physician uncertainty, the AUC for post-AI predictions (0.694, 95% CI 0.642–0.746) was significantly higher than that for pre-AI predictions (0.610, 95% CI 0.560–0.660, p-value < 0.001; Table 3). The Sankey diagrams in Fig. 4 show the changes in physician predictions according to the physician confidence level and AI-BPM prediction results.

**Table 3:** Discrimination performance of physicians before and after the use of the AI-BPM in the sampled dataset (risk threshold: 5%).

Dataset	AUC (95% CI)	p-value	Sensitivity (95% CI)	p-value	Specificity (95% CI)	p-value
Total, N = 1,000						
AI-BPM	0.770 (0.726–0.815)	–	0.933 (0.874–0.966)	–	0.332 (0.301–0.364)	–
Pre-AI	0.639 (0.585–0.693)	Reference	0.839 (0.759–0.920)	Reference	0.309 (0.252–0.365)	Reference
Post-AI	0.703 (0.654–0.753)	< 0.001	0.904 (0.858–0.950)	0.02	0.310 (0.262–0.358)	0.92
Subgroups						
AI-BPM uncertainty						
Low, n = 585						
AI-BPM	0.794 (0.747–0.842)	–	0.978 (0.923–0.994)	–	0.321 (0.282–0.364)	–
Pre-AI	0.649 (0.594–0.704)	Reference	0.865 (0.790–0.940)	Reference	0.298 (0.238–0.358)	Reference
Post-AI	0.710 (0.661–0.759)	< 0.001	0.938 (0.900–0.976)	0.01	0.303 (0.249–0.357)	0.77
High, n = 415						
AI-BPM	0.616 (0.510–0.723)	–	0.800 (0.627–0.905)	–	0.345 (0.300–0.394)	–
Pre–AI	0.567 (0.451–0.682)	Reference	0.739 (0.544–0.934)	Reference	0.323 (0.256–0.390)	Reference
Post-AI	0.593 (0.498–0.688)	0.24	0.783 (0.649–0.917)	0.33	0.320 (0.269–0.371)	0.86
Physician uncertainty						
Low, n = 213						
AI-BPM	0.772 (0.677–0.867)	–	0.903 (0.751–0.967)	–	0.341 (0.276–0.412)	–
Pre-AI	0.720 (0.640–0.801)	Reference	0.851 (0.758–0.944)	Reference	0.451 (0.336–0.565)	Reference
Post-AI	0.762 (0.674–0.850)	0.05	0.901 (0.813–0.989)	0.19	0.433 (0.324–0.542)	0.32
High, n = 787						
AI-BPM	0.770 (0.720–0.820)	–	0.944 (0.875–0.976)	–	0.330 (0.296–0.365)	–
Pre-AI	0.610 (0.560–0.660)	Reference	0.850 (0.770–0.930)	reference	0.259 (0.194–0.324)	Reference
Post-AI	0.694 (0.642–0.746)	< 0.001	0.917 (0.865–0.970)	0.01	0.268 (0.210–0.326)	0.48

AUC, area under the receiver operating characteristic curve; CI, confidence interval; AI, artificial intelligence.

The reliability of the pre-AI predictions between two physicians showed a minimal level of agreement using Cohen’s kappa statistic (κ = 0.28), but was increased in the post-AI predictions (κ = 0.38). In the post-experiment survey using a 5-point Likert scale (1: strongly disagree, 5: strongly agree), the participating physicians rated an average of 4.1 (SD: 0.7) points for the statement “Providing explanations of the AI model’s predictions increased the trustworthiness of the model.” Additionally, the participating physicians rated an average of 4.1 (SD: 0.9) points for the statement “Providing confidence intervals for the AI model’s predictions increased the trustworthiness of the model”.

## Discussion

In this two-phase study, we first developed and validated an AI-BPM and subsequently examined its impact on physician predictions using historical patient records. The proposed AI-BPM is a BNN-based multi-modal prediction model that utilizes both structured and unstructured text data available at the early stage of an ED visit and was developed and validated using large datasets. Temporal and external validation of the AI-BPM indicated acceptable discrimination and calibration performance, with AUCs for predicting bacteremia in the range of 0.73–0.76. In the validation datasets, the sensitivities and specificities at a threshold of 5% were in the ranges of 0.91–0.93 and 0.31–0.34, respectively. When the AI-BPM was used as a CDSS, the physician performance of predicting bacteremia was significantly improved. The AUC increased from 0.64 to 0.70 and the sensitivity increased from 0.84 to 0.90 after utilizing the AI-BPM. The predictive performance of physicians was especially improved in cases where they had low confidence in their predictions (high physician uncertainty) and the AI-BPM had high confidence (low AI-BPM uncertainty). The strengths of this study include a large sample size, development of a novel AI bacteremia prediction model that considers the uncertainties of its predictions, and validation of the model on an external dataset. Additionally, to the best of our knowledge, this study is one of the first to explore the impact of an AI model on physicians considering the uncertainties of both the physician and the AI model.

A recently published study compared physician gestalt with two well-established prediction models for predicting bacteremia^13^. In the study, the AUC and sensitivity (at a 5% risk threshold) of predicting bacteremia using physician gestalt were 0.79 and 0.97, respectively, which are higher than those of the pre-AI physician predictions reported in our study. However, there are significant differences in the setting: the predictions made using physician gestalt in the previous study were performed just before admission and were therefore based on information already obtained, including imaging and laboratory tests. Because such time-consuming information is often not available at the time ED blood cultures are performed, the study’s results do not truly reflect the performance of physician gestalt to avoid unnecessary blood cultures in the ED.

Several validated bacteremia prediction models that use laboratory test results as inputs have demonstrated an AUC of 0.74–0.75 in the ED setting^13,21,22^. The AI-BPM, without using laboratory test results, achieved comparable performance with existing prediction models by utilizing multi-modal data. Natural language processing was used to mine unstructured clinical notes to enable early prediction of sepsis in a previous study^25^. However, to our knowledge, there is currently no bacteremia prediction model that incorporates unstructured text data. We believe that a multi-modal AI model that integrates such data greatly enhances the ability to formulate early and accurate predictions. The important features of the AI-BPM for predicting bacteremia included old age, fever, hypotension, and history of chills, which are similar to previous studies^21,22^. Words including “sputum”, “cough”, and “dyspnea” decreased the predicted probability of bacteremia, which is consistent with previous findings that found a low prevalence of bacteremia in patients with respiratory tract infections^26^.

The uncertainty of AI model predictions can be assessed in two ways: by analyzing the point estimate or the dispersion of the estimate^27^. When a prediction’s point estimate is very low or high, it can be considered highly confident, while an estimate in the middle range may indicate less confidence in a binary classification problem. BNNs are particularly effective at capturing the second type of uncertainty, in which a narrow CI suggests high confidence and a wide CI suggests low confidence. This study’s approach to defining AI uncertainty encompasses both types of uncertainties mentioned above. Specifically, whether the risk threshold value (5%) falls within the 95% CI of the AI-BPM prediction is determined by both the point estimate and the dispersion of the prediction. This definition of AI uncertainty also considers clinical knowledge. For instance, an AI model prediction yielding a point estimate of 0.5 with a 95% CI of 0.3–0.7 might suggest a high degree of uncertainty in some situations; however, a model designed to predict the probability of bacteremia would still confidently recommend to the physician that blood cultures be performed.

In our study, physician uncertainty was assessed based on their subjective confidence in their predictions. This measure may be influenced by various factors, such as the physician’s clinical experience and personality and the patient information provided. For instance, a physician may be confident in predicting the absence of bacteremia using gestalt for a young healthy patient with specific symptoms suggestive of upper respiratory infection. However, predicting bacteremia in a patient with vague symptoms can be challenging. The AI-BPM has demonstrated significant value in situations with such high physician uncertainty.

The results of this study indicate that the physician—AI interaction process closely resembles the traditional clinical decision-making process. When faced with high levels of uncertainty, a physician may seek advice from a peer or obtain further diagnostic test results. The likelihood of the physician accepting recommendations may be higher if the peer is experienced or if the diagnostic test results are definitive. An AI prediction model with suitable performance can potentially serve as either an experienced peer or a valuable diagnostic test. In this context, providing the level of uncertainty and explanations for the prediction are crucial to ensure physicians will trust the AI model^2^.

One noteworthy finding of this study is that the use of AI-BPM improved the sensitivity of physicians, while the specificity remained unchanged. This is likely because physicians prioritize safety over other factors due to the severe consequences of missing life-threatening conditions^13^. Another interesting discovery was that the AI-BPM diagnostic performance was similar in subgroups with low and high physician uncertainty, which suggests that the AI-BPM may be interpreting clinical information differently from physicians, thus enabling it to perform well even in situations where physicians lack confidence.

There were 18 cases of bacteremia in which the physician initially assessed the risk of bacteremia as very low but subsequently revised their evaluation to a higher risk after utilizing AI-BPM. Among these cases, 10 did not display fever upon presentation at the ED and lacked any documentation of fever or chills in the physician's notes. The patients were elderly (with a mean age of 70.8 years) and exhibited symptoms such as abdominal pain, headache, dyspnea, hematemesis, and altered mental state. These findings highlight the significance of this study in medical education, as it identifies scenarios where physicians may exhibit weaknesses in predicting bacteremia. Addressing these areas of weakness through appropriate training can improve diagnostic accuracy and patient care.

This study has several limitations. First, we used data from academic tertiary hospitals located in urban areas, which may limit the generalizability of this study. The characteristics of patients and the decision criteria to obtain blood cultures may be different in other settings. Second, Phase 1 of the study used retrospectively collected data, which could potentially include unmeasured biases. Third, we assessed the impact of the AI model on physicians using historical patient records instead of evaluating it in the real-world setting. Therefore, the reviewing physicians were not able to examine the patients themselves, but were only able to read the examination results from the historical patient record. The completeness and accuracy of the physician notes may have also affected the study results. Fourth, reading order bias may have been involved due to the sequential reading design of this study^28^. However, a sequential reading design was also adopted in many previous studies, and it was necessary to evaluate the prediction changes of physicians according to their uncertainty^29,30^. Finally, although we did not specifically enroll physicians who either favored or opposed the adoption of AI models, the participating physicians’ familiarity with and attitude towards AI may have influenced the impact of the AI-BPM.

This study provides several important insights into the factors that should be considered during the process of AI model implementation in the healthcare system. First, the uncertainty of the physicians, which is associated with the effectiveness of a novel AI model implementation, should be considered. An AI model would be of greater utility if it can provide accurate predictions in clinical situations where physicians are highly uncertain. Additionally, the baseline predictive performance of the physicians should be measured and reported to the physicians. If physicians are unaware of their baseline predictive performance, they can become overconfident, which may lead to decreased effectiveness of AI model implementation^31^. Second, AI model prediction uncertainty should be considered to allow physicians to make proper decisions in tandem with the model. For example, in the bacteremia prediction setting of our study, a predicted probability of 0.08 (95% CI 0.03–0.13) would indicate an uncertain prediction, whereas a model that considers only the point estimate (0.08) would simply recommend performing blood cultures. Finally, satisfactory explanations and estimates of prediction uncertainty should be provided to acquire the physicians’ trust and enable effective AI model implementation^11^.

In conclusion, the AI-BPM, a BNN-based model that captures the uncertainty of its predictions, was developed and externally validated. The use of the AI-BPM significantly improved the predictive performance of physicians, especially in cases where physicians were uncertain and the AI-BPM was confident. Although further clinical trials are necessary to assess the effectiveness of the AI-BPM in real-world clinical settings, our study provides insight into the potential benefits of physician–AI model collaboration in enhancing predictive accuracy in uncertain clinical tasks.

## Methods

### Study design and setting

Cases of ED visits to Seoul National University Hospital (Hospital A) between January 2016 and December 2017 were used for AI-BPM development. ED visits to Hospital A between January 2018 and December 2018 were used for temporal validation. Cases of ED visits to Seoul National University Bundang Hospital (Hospital B) between January 2018 and December 2018 were used for external validation (Fig. 1). Hospitals A and B have annual ED visits of 70,000–90,000 and receive both referred patients and patients from the regional community. Data, including patient demographics, vital signs, symptoms, ED physician notes, and ED outcomes, were extracted from the clinical data warehouses of the study institutions.

A graphical user interface (GUI) was developed to simulate an electronic medical record (EMR) system that presents a patient’s baseline characteristics (age and sex), ambulance use, ED triage level, initial vital signs, mental status, and initial ED physician notes (Supplementary Fig. 2). The GUI depicted an EMR of a recently arrived ED patient who had just been examined by an ED physician. Historical records of the patients in the sampled dataset were used. Before the study, the participating physicians were briefly informed of the AI-BPM development process and the predictive performance of the AI-BPM in the development dataset. The physicians reviewed the records in the GUI and selected the estimated probability of bacteremia on an ordinal scale (very low, 0–5%; low, 5–10%; low–moderate, 10–20%; moderate 20–50%; high, 50–100%) using clinical gestalt (pre-AI prediction). The ordinal scale of bacteremia probability was determined according to a previous review^26^. They also chose the confidence level of their predictions on a 5-point Likert scale (1, very low; 2, low; 3, moderate; 4, high; 5, very high) for each of the patients. After a pre-AI prediction was made, the AI-BPM prediction of bacteremia probability along with its 95% CIs were presented on the GUI sequentially. Additionally, the local feature importance using SHapley Additive exPlanations (SHAP) was shown as a bar plot on the GUI to inform the reviewing physician how each variable influenced the output of the AI-BPM for each case^32^. The physicians were asked to rerate the probability of bacteremia and the confidence level of their predictions after observing the results of the AI-BPM (post-AI prediction).

### Study population

All adults (aged ≥ 18 years) who visited the ED of the study institutions during the corresponding study period and had at least two sets of blood cultures taken during their ED stay were included. Different ED visits from the same patient were considered as separate cases. Cases without matching ED physician notes were excluded. The decision to obtain blood cultures was made by the attending ED physician, similar to the process in previous studies^13,21^.

### Variables and measurements

Both structured and unstructured data were used as inputs for the AI-BPM. Structured data including age, SBP, DBP, HR, respiratory rate, and BT as continuous variables and sex, ambulance use, injury-related visit, referred, ED triage level (levels 1–5), mental status (alert/verbal/pain/unresponsive), history of chills, vomiting, and abdominal pain as categorical variables were collected. Variables with significant difference between patients with bacteremia and those without bacteremia in the development dataset were used as predictors for the AI-BPM (Supplementary Table 1). Vital signs, mental status, and ED triage level were measured by the triage nurse shortly after a patient’s arrival to the ED. The ED triage level was determined by the Korean Triage and Acuity Scale, which was developed based on the Canadian Triage and Acuity Scale^33^. The symptoms of patients were recorded by the initial attending ED physician. While there were some missing vital sign data in all three datasets, the proportions of data missing were less than 3%. Missing data were imputed with mean values. Other variables excluding vital signs had no missing data. Continuous variables were standardized to zero mean and unit variance. Categorical variables were one-hot encoded.

A patient’s present illness and past medical history recorded by the initial attending ED physician were used as unstructured data for the AI-BPM. The notes were documented immediately after the attending ED physician examined the triaged patient. Physician notes were written in bilingual (English/Korean) free-text format, which is a common practice in Korea^34^. Text preprocessing, including removal of punctuation marks, deleting English and Korean stop words, substituting capital letters with lowercase, and lemmatization, was performed. Subsequently, each note was vectorized using the term frequency–inverse document frequency (TF–IDF) vectorizer with the minimum document frequency set to 1%. The TF–IDF method was chosen for this study because it offers several advantages, including the ability to manage bilingual text, ease of interpretation, and comparable performance to more complex algorithms^35,36^. The full list of predictors used in the AI-BPM are presented in Supplementary Table 3.

### Development of the AI-BPM

The development dataset was randomly split into two for hyperparameter tuning, in which 80% of the data were used for AI-BPM training and the remaining 20% were used for validation. Subsequently, the AI-BPM was trained on the entire development dataset with the optimal hyperparameters (Supplementary Table 4). The BNN algorithm is a type of neural network with Bayesian inference. The AI-BPM, which is based on the BNN algorithm, receives two inputs: preprocessed structured data and vectorized encoding based on TF–IDF from unstructured text data. The structured data input and TF–IDF vector input were connected to hidden layers of 100 and 15 nodes, respectively. The hidden layers were concatenated and then connected to a single output node. All layers were densely connected and used the Flipout estimator for Bayesian variational inference^37^. While a standard neural network is trained to find the point estimates of the weights and outputs, a BNN is trained to find the marginal distributions of the weights and outputs that best fit the data^38^. Because the AI-BPM is based on BNN, the uncertainty of each of the predictions can be estimated^9,38^. To calculate the mean and SD of the AI-BPM output distribution for a single patient case, 25 samples are taken from the output distribution. The final prediction of the AI-BPM is then determined as the mean of the output distribution. The 95% CI, derived from the SD, is used to define the uncertainty of the AI-BPM prediction.

### Definition of bacteremia

The definition of bacteremia and the process of obtaining blood cultures are described in our previous study^15^. In brief, bacteremia is defined as the growth of a pathogenic bacteria (excluding common commensals defined by the National Healthcare Safety Network guideline) in at least one blood culture. For each set of blood cultures, 10 cc of blood was drawn from different venipuncture sites.

### Study outcomes

The primary outcome of this study was the AUC for prediction of bacteremia. The secondary outcomes were sensitivity and specificity for prediction of bacteremia. According to previous literature, blood cultures may not be necessary for patients with a predicted bacteremia probability of less than 5% or 10%^13,26^. In our study, we analyzed the results of Phase 1 using risk thresholds of 5% and 10%. In other words, the estimated risk obtained from the output of the AI-BPM was binarized into positive or negative predictions according to the threshold of 5% or 10%. However, we found that the AI-BPM sensitivity for predicting bacteremia was less than 0.80 when the 10% threshold was used. This low sensitivity may not be acceptable, given that undetected bacteremia in the ED can be fatal. Therefore, we conducted the analysis of Phase 2 using a risk threshold of 5% only.

### Statistical analysis

Categorical variables were reported as numbers and proportions, and the chi-square test was used for comparisons between groups. Continuous variables were reported as means and SDs, and the Student’s t-test was used for comparisons between groups. A two-sided p-value less than 0.05 was considered statistically significant. All statistical analyses were performed using Python version 3.8.12 (Python Software Foundation, Wilmington, DE, USA) and R version 3.6.3 (RStudio, Boston, MA, USA).

In Phase 1, the discrimination performance of the AI-BPM in each dataset was assessed using AUC, sensitivity, specificity, positive predictive value, negative predictive value, and their CIs, which were obtained using DeLong’s method^40^. The calibration of the AI-BPM was assessed using the calibration plot. The global feature importance of the AI-BPM was obtained using mean absolute SHAP values^32^. Additionally, we conducted an ablation study in which we assessed the discrimination performance of two additional models: one using structured data only and another using unstructured data only to predict bacteremia. The purpose of this study was twofold: firstly, to evaluate the individual contribution of structured and unstructured data to the model's performance, and secondly, to account for scenarios where both types of data might not be available in some hospitals. The architectures of the models were slightly modified from the AI-BPM so that they would only utilize the layers corresponding to the type of data they were using (Supplementary Table 4).

In Phase 2, the reviewing physician pre- and post-AI AUC, sensitivity, and specificity for predicting bacteremia were calculated and compared using the Obuchowski–Rockette method to account for the “multiple readers of multiple cases” design (https://cran.r-project.org/package=MRMCaov)^41,42^. The average receiver operating characteristics curve from multiple reviewing physicians was presented^43^. The physician pre- and post-AI confidences on a Likert scale were compared using the paired t-test. The inter-rater reliability between two physicians was assessed using linearly weighted Cohen’s kappa statistic^44^. Subgroup analysis of the sampled dataset was performed according to two types of uncertainties: AI-BPM uncertainty and physician uncertainty. High AI-BPM uncertainty was defined as when the threshold value (5%) was included in the 95% CI of the AI-BPM prediction, and low AI-BPM uncertainty was when it was not included. To be detailed, although the inherent uncertainty of BNN is represented as CI, the uncertainty of the AI-BPM was redefined as whether the CI encloses the risk threshold. High physician uncertainty was defined as when at least one of the two reviewing physicians scored confidence below 4 points, while low physician uncertainty was when both physicians scored confidence 4 points or higher^45^.

### Ethics statements

This study was approved by the Institutional Review Board of Seoul National University Hospital (No. 2212-167-1393). Need for informed consent from patients was waived by the Institutional Review Board of Seoul National University Hospital for both Phase 1 and 2 due to the retrospective nature of patient data collection. Written informed consent was obtained from the 20 participating physicians in Phase 2. The study protocol adhered to the ethical guidelines of the 1975 Declaration of Helsinki and its subsequent revisions. We followed the Transparent Reporting of a Multivariable Prediction Model for Individual Prognosis or Diagnosis (TRIPOD) guidelines on reporting the study results.

### Supplementary Information

Supplementary Information.

## Data Availability

The raw data used in this study are not publicly available because they contain individual patients’ information and their medical records. However, deidentified data excluding personal information and medical records may be available from the corresponding author upon reasonable request.
