# Supplementary material for: Development of an artificial intelligence bacteremia prediction model and evaluation of its impact on physician predictions focusing on uncertainty
Source: Sci Rep. 2023 Aug 19;13:13518. doi: 10.1038/s41598-023-40708-2 (PMC10439897; doi:10.1038/s41598-023-40708-2)

Title: Development of an artificial intelligence bacteremia prediction model and evaluation of its impact on physician predictions focusing on uncertainty

Authors: Dong Hyun Choi, Min Hyuk Lim, Ki Hong Kim, Sang Do Shin, Ki Jeong Hong*, Sungwan Kim*

*Corresponding authors: Sungwan Kim (e-mail: sungwan@snu.ac.kr) and Ki Jeong Hong (e-mail: emkjhong@gmail.com)

Supplementary Table 1. Characteristics and outcomes of patients with and without bacteremia in the development dataset

	With bacteremia (N = 1,670)	Without bacteremia (N = 13,692)	p-value
Demographics			
Age, years	66.3 (12.5)	61.8 (16.2)	<0.001
Sex, male	935 (56.0)	7,498 (54.8)	0.34
Ambulance use	760 (45.5)	4,736 (34.6)	<0.001
Referred from other hospital	357 (21.4)	3,308 (24.2)	0.01
Injury related visit	11 (0.7)	124 (0.9)	0.31
ED triage level			<0.001
Level 1	129 (7.7)	662 (4.8)	
Level 2	471 (28.2)	3,546 (25.9)	
Level 3	950 (56.9)	8,022 (58.6)	
Level 4	119 (7.1)	1,447 (10.6)	
Level 5	1 (0.1)	15 (0.1)	
Initial mental status			0.004
Alert	1,471 (88.1)	12,423 (90.7)	
Verbal	151 (9.0)	922 (6.7)	
Pain	34 (2.0)	251 (1.8)	
Unresponsive	14 (0.8)	96 (0.7)	
Initial vital signs			
SBP, mmHg	129.4 (29.8)	137.0 (27.9)	<0.001
DBP, mmHg	71.9 (15.1)	77.1 (15.0)	<0.001
HR, mmHg	104.3 (20.8)	99.0 (19.9)	<0.001
RR, mmHg	19.9 (4.4)	19.8 (4.4)	0.61
BT, ℃	37.7 (1.3)	37.3 (1.1)	<0.001
Symptom history			
Chills	599 (35.9)	3,193 (23.3)	<0.001
Vomiting	259 (15.5)	1,339 (9.8)	<0.001
Abdominal pain	385 (23.1)	2,246 (16.4)	<0.001
Outcomes			
Hospital admission	1,252 (75.0)	7,660 (55.9)	<0.001
Death in ED	10 (0.6)	28 (0.2)	0.002

Categorical variables are presented as numbers (proportions) and continuous variables are presented as means (standard deviations). Hospital admission included patients admitted to the ward or intensive care unit. Abbreviations: ED, emergency department; SBP, systolic blood pressure; DBP, diastolic blood pressure; HR, heart rate; RR, respiratory rate; BT, body temperature.

Supplementary Table 2. Discrimination performance for predicting bacteremia according to variables utilized (risk threshold: 5%)

Dataset	AUC (95% CI)	Sensitivity (95% CI)	Specificity (95% CI)	PPV (95% CI)	NPV (95% CI)
Structured data only					
Development	0.718 (0.705–0.731)	0.925 (0.912–0.937)	0.256 (0.248–0.263)	0.132 (0.126–0.138)	0.966 (0.959–0.971)
Temporal validation	0.703 (0.684–0.721)	0.928 (0.909–0.944)	0.258 (0.248–0.268)	0.126 (0.118–0.135)	0.969 (0.960–0.976)
External validation	0.679 (0.660–0.697)	0.923 (0.904–0.938)	0.202 (0.192–0.212)	0.154 (0.145–0.164)	0.943 (0.929–0.954)
Unstructured data only					
Development	0.751 (0.740–0.763)	0.960 (0.949–0.968)	0.261 (0.254–0.269)	0.137 (0.131–0.143)	0.982 (0.977–0.985)
Temporal validation	0.679 (0.660–0.698)	0.936 (0.918–0.951)	0.174 (0.165–0.183)	0.116 (0.108–0.123)	0.959 (0.947–0.969)
External validation	0.681 (0.663–0.699)	0.944 (0.927–0.956)	0.191 (0.182–0.201)	0.155 (0.146–0.165)	0.956 (0.942–0.966)

Abbreviations: AUC, area under the receiver operating characteristic curve; CI, confidence interval; PPV, positive predictive value; NPV, negative predictive value.

Supplementary Table 3. Description of predictors used for the AI-BPM

Variable	Data type	Number of variables after preprocessing
Structured data		17
Age	Continuous	1
Ambulance use	Binary	1
Referred	Binary	1
ED triage level	Categorical*	4
Initial mental status	Categorical*	3
Systolic blood pressure	Continuous	1
Diastolic blood pressure	Continuous	1
Heart rate	Continuous	1
Body temperature	Continuous	1
History of chills	Binary	1
History of vomiting	Binary	1
History of abdominal pain	Binary	1
Unstructured data (Vectorized encoding based on TF-IDF)		590

Abbreviations: ED, emergency department; TF-IDF, term frequency-inverse document frequency.
*One hot encoding into N-1 variables were performed for categorical data.

Supplementary Table 4. The structure and optimal hyperparameters of the AI-BPM

	Value
Input layer 1 (structured data)	Number of nodes: 17
Input layer 2 (Vectorized encoding based on TF-IDF)	Number of nodes: 590
Hidden layer 1 (connected with input layer 1)	Number of nodes: 100, activation function: Rectified Linear Unit
Hidden layer 2 (connected with input layer 2)	Number of nodes: 15, activation function: Rectified Linear Unit
Output layer	Number of nodes: 1, activation function: sigmoid
Hyperparameter related to training	Epochs: 15, batch size: 20, optimizer: Adam, learning rate: 0.0002

Abbreviations: TF-IDF, term frequency-inverse document frequency

Input layer 1 and Hidden layer 1 were not used in the model utilizing unstructured data only.

Input layer 2 and Hidden layer 2 were not used in the model utilizing structured data only.

Supplementary Figure 1. The global feature importance of the AI-BPM

Legend: For a single case, a positive SHAP value indicates that the feature pushed the output of the AI-BPM towards predicting bacteremia, whereas a negative SHAP value indicates that the feature pushed the output of the AI-BPM towards predicting no bacteremia. Each dot of the diagram represents a single case. Features with (Eng) and (Kor) are English and Korean words from the ED physician notes. Abbreviations: HR, heart rate; BT, body temperature; DBP, diastolic blood pressure; SBP, systolic blood pressure; ERBD, endoscopic retrograde biliary drainage; SHAP, Shapley Additive exPlanations.

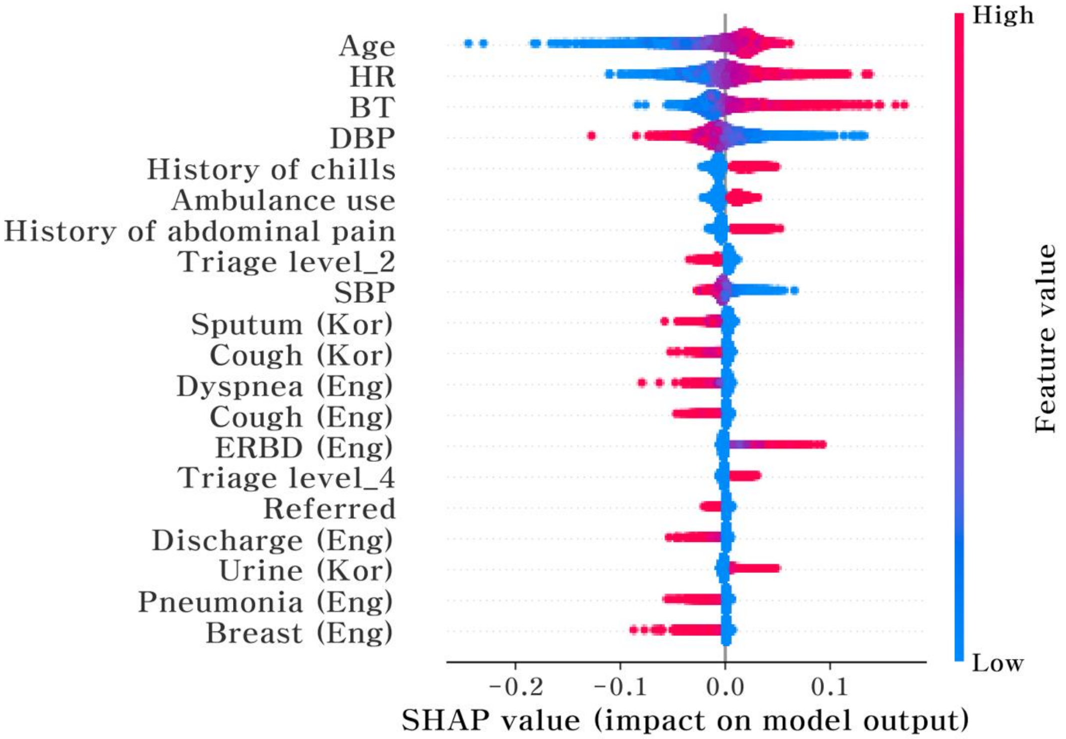

Supplementary Figure 2. Graphical user interface used in this study **a** before and **b** after AI-BPM prediction results are shown.

Legend: The patient's medical records are blanked out for protection of personal information.

a

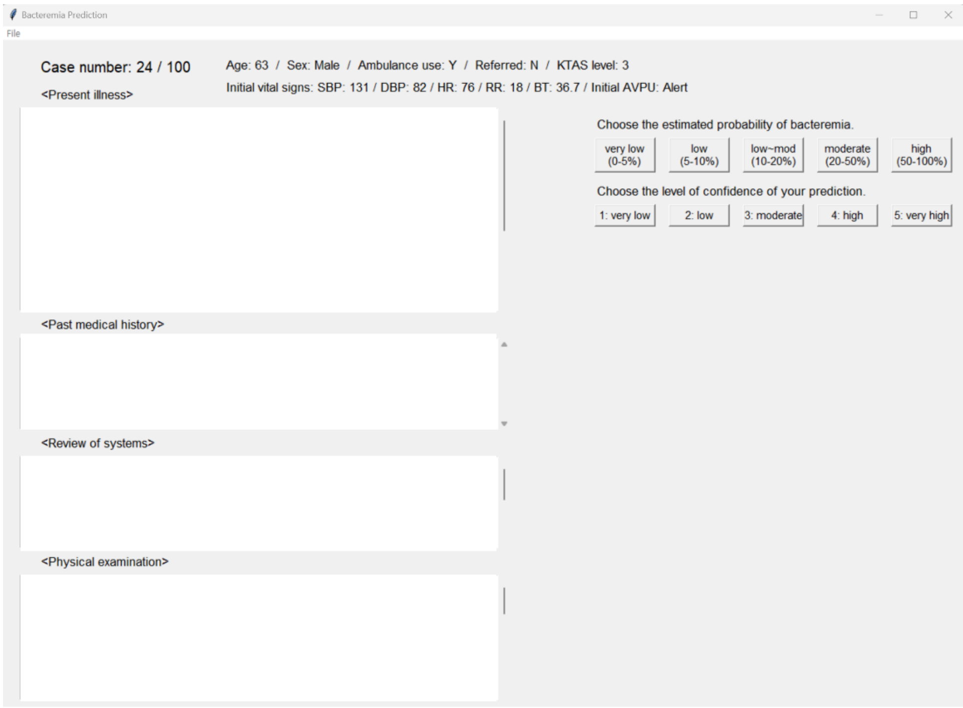

b

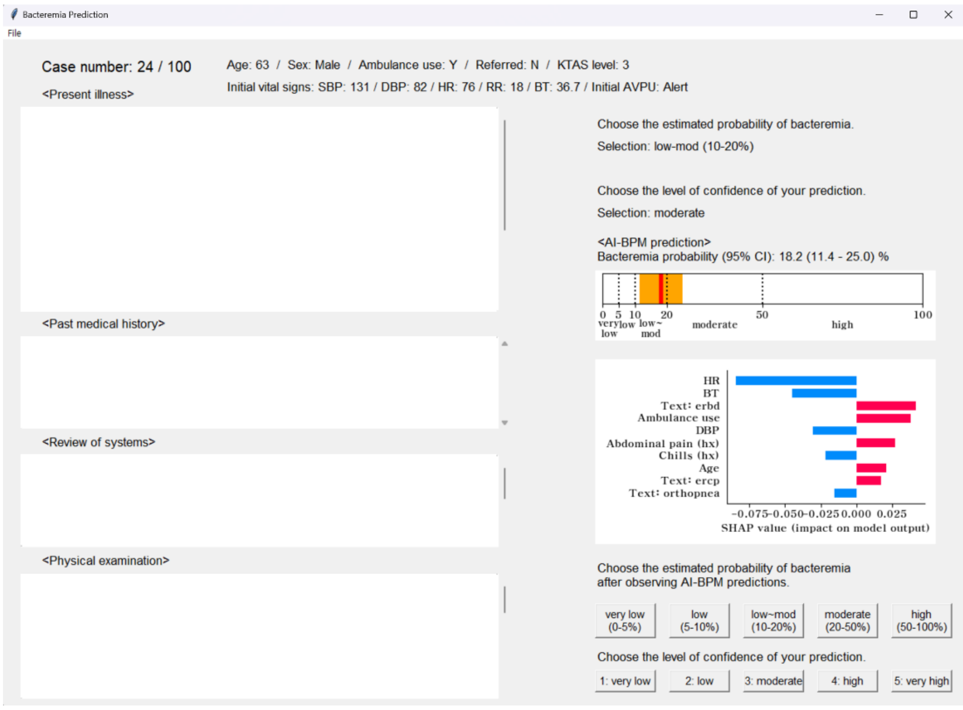

Supplement: Supplementary file 1 — Supplementary Information. [file 41598_2023_40708_MOESM1_ESM.pdf]
